# Ultra-rapid object categorization in real-world scenes with top-down manipulations

**DOI:** 10.1371/journal.pone.0214444

**Published:** 2019-04-10

**Authors:** Bingjie Xu, Mohan S. Kankanhalli, Qi Zhao

**Affiliations:** 1 NUS Graduate School for Integrative Sciences and Engineering, National University of Singapore, Singapore, Singapore; 2 School of Computing, National University of Singapore, Singapore, Singapore; 3 Department of Computer Science and Engineering, University of Minnesota, Minneapolis, MN, United States of America; Universitat Hamburg, GERMANY

## Abstract

Humans are able to achieve visual object recognition rapidly and effortlessly. Object categorization is commonly believed to be achieved by interaction between bottom-up and top-down cognitive processing. In the ultra-rapid categorization scenario where the stimuli appear briefly and response time is limited, it is assumed that a first sweep of feedforward information is sufficient to discriminate whether or not an object is present in a scene. However, whether and how feedback/top-down processing is involved in such a brief duration remains an open question. To this end, here, we would like to examine how different top-down manipulations, such as category level, category type and real-world size, interact in ultra-rapid categorization. We have constructed a dataset comprising real-world scene images with a built-in measurement of target object display size. Based on this set of images, we have measured ultra-rapid object categorization performance by human subjects. Standard feedforward computational models representing scene features and a state-of-the-art object detection model were employed for auxiliary investigation. The results showed the influences from 1) animacy (*animal*, *vehicle*, *food*), 2) level of abstraction (*people*, *sport*), and 3) real-world size (four target size levels) on ultra-rapid categorization processes. This had an impact to support the involvement of top-down processing when rapidly categorizing certain objects, such as *sport* at a fine grained level. Our work on human vs. model comparisons also shed light on possible collaboration and integration of the two that may be of interest to both experimental and computational vision researches. All the collected images and behavioral data as well as code and models are publicly available at https://osf.io/mqwjz/.

## Introduction

Visual recognition of objects by humans is often rapid and seemingly effortless [1–3]. Humans can accurately make judgments about briefly presented scenes, such as the presence of a target category and its referent location [1]. In particular, it is possible to reliably detect objects in the central visual field within a single fixation in less than 200 ms [3]. It is widely agreed that the human brain recognizes and differentiates objects from one another, and in a similar manner groups them into categories according to common features in a hierarchical fashion [4, 5]. The features extracted from later stages of the hierarchy are more invariant to identity-preserving transformations, such as changes in size, location, and orientation [6–9].

Object categorization is commonly believed to be achieved by interaction between bottom-up and top-down cognitive processing [10]. In the ultra-rapid categorization scenario where the stimuli appear briefly and response time is limited, it is assumed that a first sweep of feedforward/bottom-up information is sufficient to discriminate whether or not an object is present in a scene [11–13]. Meanwhile, whether and how feedback/top-down processing is involved in such a brief duration has attracted a lot of research attention [11, 13–15]. Notably, a recent work [14] based on MEG-fMRI fusion method disentangles an initial bottom-up sweep from subsequent top-down processing, and reveals the following emergence of categorical information which indexes time-consuming feedback processing. With this, here, we would like to examine how different top-down manipulations, such as category level, category type and real-world size, interact in ultra-rapid categorization.

It is known that top-down processing is required to achieve complex visual tasks, such as object identification, which are beyond pure object detection and include more detailed analyses of the object and its semantic interpretation [15–18]. Top-down influences on object categorization include spatial and feature-based attention, the likelihood of an object being present, expertise, the level of abstraction and thus the amount of information necessary to analyze the object, the object category (e.g. animacy), and the surrounding contextual information [15]. Based on these knowledge, we investigate the combination of different top-down influences on ultra-rapidly categorizing visual objects by varying the level of category and animacy sampled with various real-world size scales.

### Animacy and real-world size

One recent proposal is that the large-scale dimensions of animacy and real-world object size organize human cognitive, perceptual, and neural object representations [19–22]. At cognitive level, both of these dimensions are closely associated with how we interact with objects. Indeed, a critical function of our visual system is to identify whether something is animate, and appropriate interactions with it depending on its size. Thus, when recognizing an object, we rapidly infer whether it is an animal [19, 23, 24] and how big it is in the real world [22]. At perceptual level, features to account for animate and inanimate object categories are distinct [15, 19, 25], so as for different real-world sizes [16]. In particular, mid-level features including spatial frequencies, textural and shape information contain important cues to distinguish animals and non-animals [19, 24, 25], as well as different real-world object sizes [16]. At neural level, animate/inanimate categories have been found to engage different neural subsystems in the brain [26]. Both dimensions of animacy and real-world size collaboratively structure cortical responses to objects. Responses to big objects (e.g. airplanes), small objects (e.g. cup cakes) and animals exhibit a tripartite organization which is mapped to the lateral and the ventral surfaces of the cortex [21].

Animal and vehicle are two broad categories commonly used to investigate effect of animacy [15, 19]. Apart from these two, among inanimate categories, food also attracts research attention which is of significant social impact [27–29]. This socially and biologically important object category is suggested to be predicted with specialized neural circuitry [29].

How the combination of top-down effects from broad animate/inanimate object categories and different real-world sizes affects human ultra-rapid behavior is still an open question [15]. Here, we focus on three broad categories: *animal*, *vehicle* and *food*, to evaluate top-down effects of animacy and real-world sizes for ultra-rapid categorization.

### Category level

Objects can be categorized at different levels of abstraction, from superordinate (e.g. animal), basic (e.g. dog) to a subordinate level (e.g. Labrador) [30]. The effect of category level reveals a cognitive process where information is evaluated corresponding to task demands. Advantage of recognition speed has been validated for superordinate categories, such as animal and vehicle [15], but not for recognition accuracy. The question arises whether this advantage generally holds for rapid categorization processes.

Here, we generalize to behavioral differences between *people* (superordinate level) and *person playing sport* denoted as *sport* (a finer grained level) in ultra-rapid categorization scenario, with built-in measurements of object sizes. Human faces [31] and bodies [32–34] are key semantic features to recognize person and sports. These two components have been reported to hold special status in perceptual processing—fast and accurately, possibly due to their social importance [31, 34]. While similarities have been reported between perceptual processes underlying human faces and bodies [35], differences in the time course of face and body perception have also been explored [33]. Therefore, we can evaluate the top-down effect from levels of abstraction thus the amount of necessary information in association with specific features.

### Contributions

**Fig 1:**
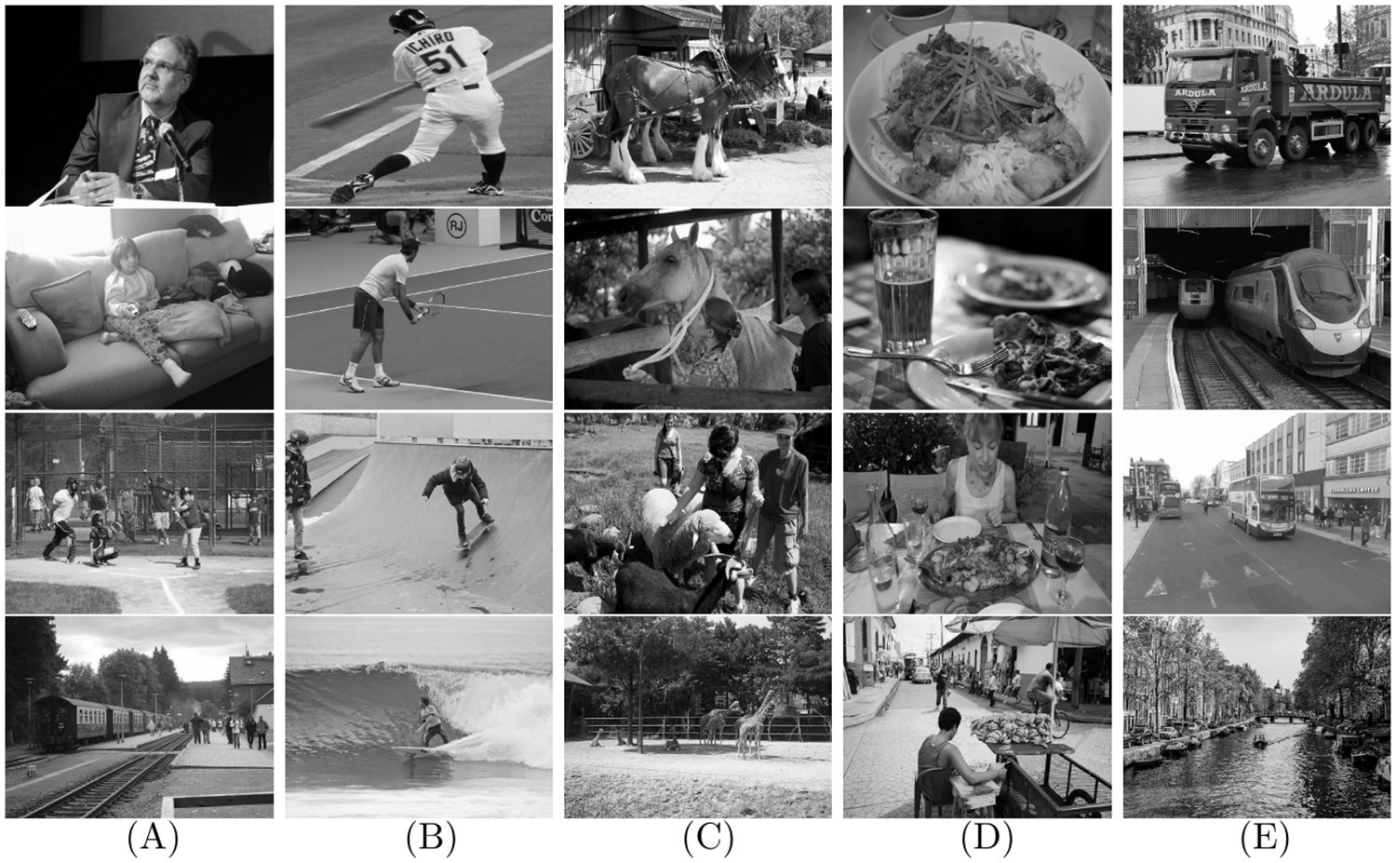
Example stimuli displayed with grayscale. Columns display target categories from: (A) *people*, (B) *sport*, (C) *animal*, (D) *food*, and (E) *vehicle*. They were characterized at four levels of target object scale (rows) that are matched across categories. The images were from the public available MS-COCO dataset from http://mscoco.org. All stimuli were allowed to be displayed to public in the original dataset. In each block with one target category, half of the stimuli were with target object present, the other half consisted of images from other four sets with different categories as targets and with this target category absent. The average luminance and contrast were equalized across the stimuli set.

To explore the combination of different top-down influences during ultra-rapid object categorization, we constructed a dataset (see Fig 1 for examples) containing 480 real-world scene images with built-in measurements of target object size scales and five common target categories [36], including *people*, *sport*, *animal*, *food* and *vehicle*. Based on the collected images, we conducted human behavioral experiments in the *ultra-rapid object recognition* task [37, 38] in which the stimuli were displayed briefly and the human observers were asked to respond rapidly and accurately. In addition to this, we also performed object recognition experiments by machines on the same set of images for an auxiliary investigation. With the advent of deep neural networks (DNNs) that have been trained on copious number of images, machines can achieve and even outperform human-level performance when classifying objects in images of real-world scenes [39]. To either explore human recognition behavior or build machines with high-level performance, a number of studies have begun investigating similarities and differences between DNNs and human recognition patterns in generalization task [40, 41], synthetic visual reasoning test [42], and visual object recognition task with isolated targets [43, 44]. In contrast to the previous work, we focus on how similarly or differently the human visual system and machines work in the scenario of object categorization with various task demands. In particular, we measure how well human observers and an advanced object detector [45] cope with object recognition when broadly sampling category levels, types and sizes in real-world scene images.

**Fig 2:**
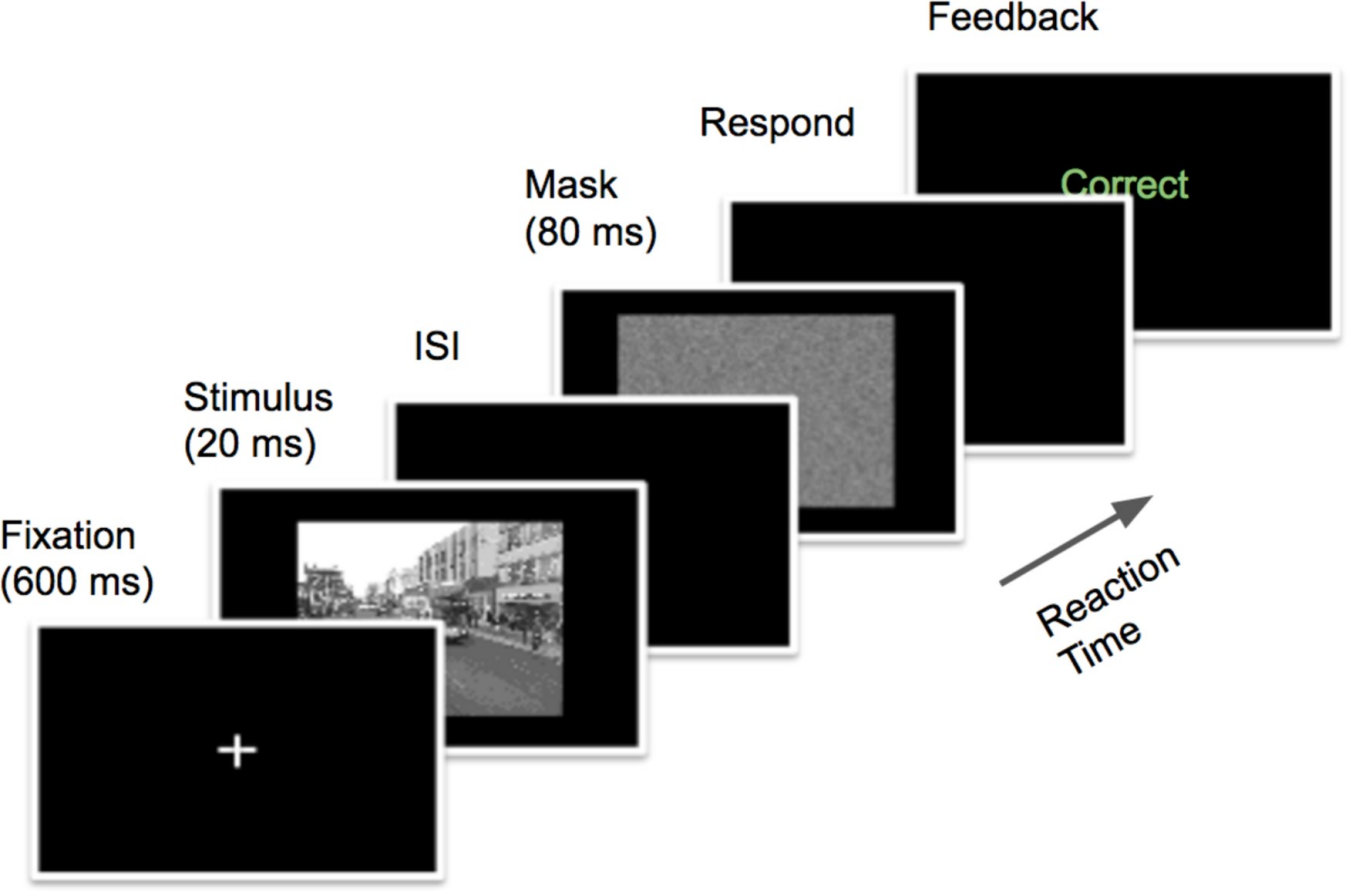
Protocol of the experiments. Subjects were tested on 96 trials in each block with one target category, organized as follows: first, the fixation cross was displayed in the center for 600 ms, then the stimulus was shown for 20 ms. After various inter-stimulus-intervals (ISIs), the random noise mask appeared, displaying for 80 ms. Four values were randomly assigned to ISI: 0, 30, 60, and 110 ms. After the mask offset, subjects were asked to respond fast and accurately within one second. The protocol was revised from the one in Bacon *et al*.’s work [37].

In the present study, we used a backward masking paradigm in ultra-rapid object recognition task [37, 38] that allowed for modulation of the accumulated amount of visual information in order to examine the time-course of processing for human performance. In this behavioral task, an image appeared for a brief duration in each presentation, and a random noise mask appeared after a controllable interval from the stimulus offset. Human subjects were required to quickly and accurately recognize whether the target category was present or absent in each presentation (i.e. image) with a key press (see Fig 2). We arranged five blocks with respective target categories, each with four levels of size scales, and evaluated recognition accuracy and speed.

## Materials and methods

### Ethics statement

All subjects gave informed written consent (as outlined in PLOS consent form) before the experiment. The experiment was approved by the NUS Institutional Review Board.

### Subjects

33 volunteers (13 female and 20 male, mean age 23, range 19-30 years) participated in the experiment. All subjects were with normal or corrected-to-normal visual acuity, 30 of them were right-handed.

### Stimuli

The data subset comprised 480 naturalistic scene images with five categories and four size scales of target objects from the publicly available Microsoft Common Object in Context (MS-COCO) dataset [36]. MS-COCO consists of naturalistic scene images with various common objects in daily context, appropriate to mimic the real-world viewing conditions. It offers 80 basic object categories in total, with diverse scales, orientations, scene complexities, and object positions. Four most common categories at superordinate level (high level of abstraction) in the MS-COCO dataset were selected: *people*, *food*, *vehicle*, *animal*, each with 96 images (Fig 1). A sub-category of *people* (i.e. people playing *sport*) was also selected to examine the effect of category level thus the amount of information necessary for ultra-rapid recognition.

Object sizes were divided into four levels based on the segmentation sizes of the objects for each target category in all MS-COCO images: *largest* if the area was larger than the first quartile of target object areas, *large* if the area was between the first and second quartile, *small* if the area was between the second and third quartile, and *smallest* otherwise. Note that the scale of *sport* category was defined by the size of the *people* playing it. Besides, we have also checked and observed that the ranges of absolute object sizes at each level are quite consistent across categories. The ranges of the absolute object size in terms of pixel amount in an image are around 0–430, 430–1,700, 1,700–7,000, 7,000–200,000 pixels from the *smallest* to the *largest* scale level, respectively (image size = 480 × 640 pixels). At each scale level that might contain a large variance of taget object size (e.g. *largest*), we have additionally matched the images with similar absolute target object sizes across categories. With that, a “large” person is similar in size as a “large” vehicle, for example. Intuitively, the scale level of a target object reflects its real-world size and its size during display, both in accordance with the scale of the other objects in the scene.

Previous images in human fast recognition studies either use isolated objects on homogeneous dark background [46], or constrain the image to only include the region around the target without a complete scene [1, 7]. In contrast to the previous stimuli, the main attributes of the current stimuli are as follows. (a) They were constructed from MS-COCO without manipulation, providing rich naturalistic scene information with diverse common objects. (b) They were selected with built-in measurement of size of target object; target objects were near the center of the images without occlusion (i.e. hand was not counted as *people*); they were with normal orientation. (c) The rich yet wild scene context allows to investigate how scene factors couple with the object recognition, just as in our daily recognition process.

All images were with resolution of 480 × 640 pixels (21^*o*^ × 16^*o*^) and displayed with grayscale against an uniform dark background with average luminance, contrast equalized across the stimuli set.

### Apparatus

The stimulus was generated using the Psychophysics Toolbox Version 3.0.12 [47] for MATLAB (Version 8.1.0, Mathworks, MA), and displayed on a LCD video monitor (24-in, refresh rate = 120 Hz, BenQ XL2420Z) controlled by a PC (3.4 GHz, Windows 7) with an GeForce GTX 770 graphics card. The display resolution was 1920 by 1080 pixels, subtending 49° by 29° at a 57-cm viewing distance (36 pixels/deg at screen center). Brightness and contrast of the monitor were adjusted to the maximum.

### Task

The task was to classify the presence/absence of the target category in naturalistic scenes with a key press. The stimulus was presented in blocks of 96 trials and organized in five blocks in total. Each block consisted of 48 trials with target category present, while the rest half absent (arranged from other four sets of targeting categories with 12 images each). In each block, trials were with the same proportion of four scales, i.e. 12 targets and 12 distractors at each scale level consistently. Note that all 480 (96 × 5) trials were arranged with different images. The sample size was determined following the previous experimental design [48]. And in each block, the distractor images were selected without any target category. At the beginning of each block, subjects were given instructions on the target category of the block. A practice session was conducted before the beginning of the experiment. The practice session was the same as in the test scenarios but with half amount of trials (48 × 5 trials). Pilot experiments were conducted to determine the practice session duration. The practice duration was chosen for subjects to coordinate their motor responses well enough, and to get familiar with the various forms of targets and distractors. It lasted around 30 minutes with breaks between blocks. Images in the practice session were not used in the test scenarios. All data in the test scenarios were used.

Specifically, each trial consisted of the following sequence of events (Fig 2). A cross for fixation was presented at the center of the screen for 600 ms, followed by the stimulus (one image) for 20 ms. A mask appeared from stimulus offset after different inter stimulus intervals (ISIs), lasting for 80 ms. ISI was randomly assigned from 0, 30, 60, 110 ms in trials within one block. The mask was the same as the one in previous work [37] that was constructed from a random noise image filtered by a Gaussian filter. After the mask offset, subjects were required to indicate whether the target category was present or not by pressing the key “F” for “yes” and “J” for “no”. They were asked to respond as quickly and accurately as possible. The maximum time limit for valid response was 1,000 ms.

### Analysis

Reaction times (RT) recorded from the offset of the mask and the proportion of correct responses were calculated. RT for only accurate trials were submitted for statistical analyses. Statistical analysis was performed by using MATLAB (Version 8.1.0, Mathworks, MA). To analyze the obtained results, repeated measures of Analysis of Variance (ANOVA), post-hoc pairwise comparisons and paired t-tests with Bonferroni correction were conducted. Error bars of graphs represent normalized 95% confidence intervals [49]. Thresholds of minimum 150 ms [3] and maximum 1000 ms were applied on RTs before the analysis for all subjects as an initial quality check.

### Computational simulation

Two computational models were employed to explain the human behavior with the identical stimuli used in human experiments: SALIENCY and GIST. In the case for SALIENCY model [50], low-level features (pixel intensity, orientations) were extracted at multiple scales and a local conspicuity map (dimension = 1200) was computed using local center-surround mechanisms for each image. Note that color feature was excluded as the images were grayscale. As for GIST model [51], global image statistics were computed by convolving the image with a Gabor filter pyramid (4 levels and 8 orientations) and further down-sampling the resulting filtered image to produce a 4 × 4 × 32 (= 512) dimensional vector, which was used for classification.

Radial basis function (RBF) kernel SVM classifiers [52] were then used for classification. A SVM was trained and tested on the same set of images as in the human behavioral experiments. Specifically, the normalized statistical image features were extracted from the training set (48 × 5 images, 50% targets and 50% distractors) and the test set (96 × 5 images, 50% targets and 50% distractors), respectively. An optimal pair of cost parameter *C* and kernel parameter *γ* was determined through grid search optimization to achieve the best performance. The grid search ranges for *C* and *γ* are [10^−1^, 10^1^] and [10^0^, 10^3^], which are empirical values. The predicted response for each test image was reported.

We also compared human recognition accuracy with the state-of-the-art object detection model. Our implementation was based on Faster R-CNN [53] with a Feature Pyramid Network (FPN) backbone built on ResNet-50. We used Tensorflow [54] for this implementation. The weights were pre-trained on ImageNet [39] and then fine-tuned on the rest of MS-COCO training set—around 35k images excluding the test images in our stimuli. This procedure used data-driven approach to mimic human visual knowledge prior about the real world, and to suit the object detection task on MS-COCO categories. The model was tested on the same set of images (96 × 5 = 480) as in human behavioral experiments.

## Results

This study investigated the influence of different 1) animacy (*animal*, *vehicle*, *food*), 2) level of abstraction (*people*, *sport*), and 3) real-world size (four target size levels) on ultra rapid object categorization.

### Animacy and level of abstraction

**Fig 3:**
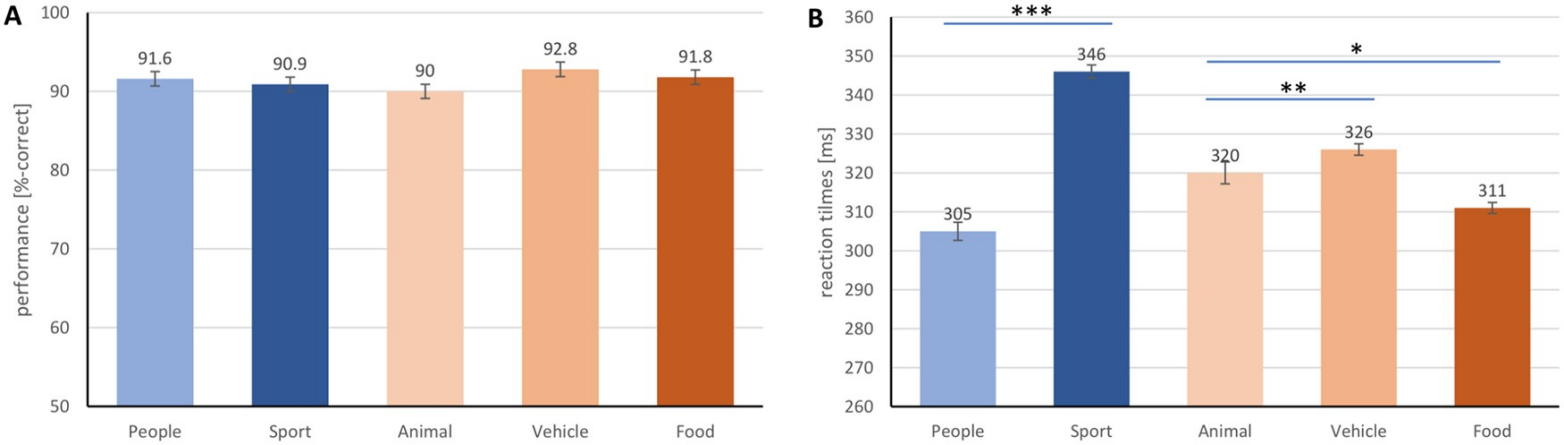
Influence of “animacy” and “level of abstraction” on ultra-rapid object categorization. Performance (A) and reaction times (B) for five category are shown. Error bars represent the normalized 95% confidence intervals [49]. *p < .05, **p < .01, ***p < .001.

We show median reaction time (RT) distribution histograms for different categories in Fig 3, and conducted analyses for effect of level of abstraction and animacy. When the task was to target *people*, the subjects were on average correct on 91.6% of the trials, with an averaged median RT of 305 ms. The recognition of *sport* involved finer grained information of the *people* interacting with it. In such case, subjects used a relatively long median RT of 346 ms and achieved accuracy of 90.9%. Significant difference was found for median RT (*p* < .001), but not for accuracies between *people* and *sport* (*p* > .05).

Regarding effect of animacy, RT from only accurate trials between targeting *animal* and the inanimate category—*vehicle* were submitted for t-tests. To recognize *animal*, *vehicle* and *food*, subjects used median RT of 320 ms, 326 ms and 311 ms, respectively. Subjects achieved respective accuracy of 90.0%, 92.8% and 91.8% (Fig 3). No significant difference of accuracy was found between *animal* and *vehicle*, neither between *animal* and *food*. But, median RTs showed difference between both pairs of animate/inanimate categories (*p* = .0075 and *p* = .0115, respectively). This supports previous studies [15, 55] investigating the effect of animacy.

### Real-world size

**Fig 4:**
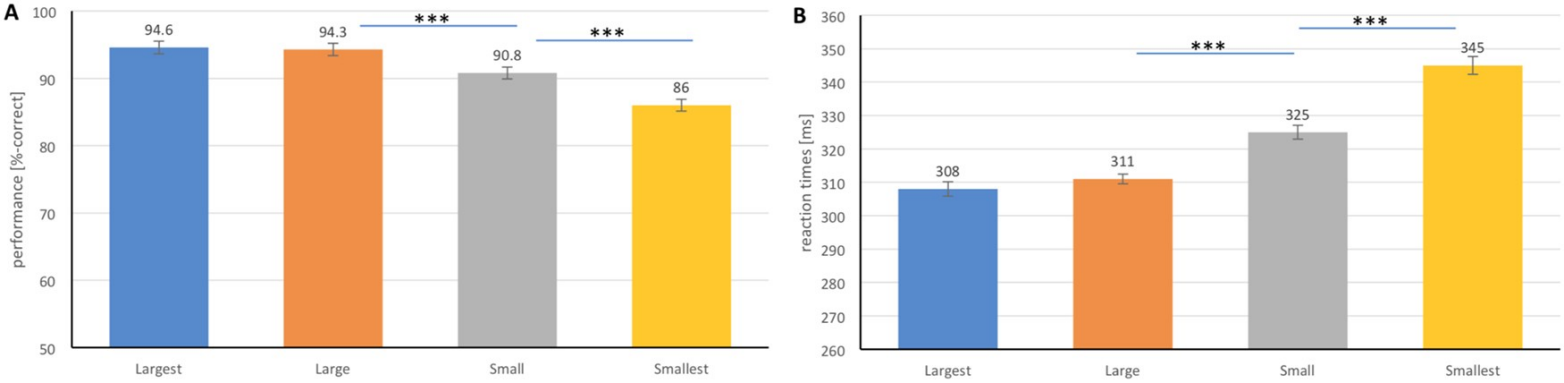
Influence of ‘‘display size level” on ultra-rapid object categorization. Performance (A) and reaction times (B) for four levels of target display size are shown. Error bars represent the normalized 95% confidence intervals [49]. *p < .05, **p < .01, ***p < .001.

From Fig 4, we can observe that with scale changed from the *largest* level to the *smallest* level, subjects on average used a longer median RT (308 ms, 311 ms, 325 ms, 345 ms), while with decreased accuracy rates (94.6%, 94.3%, 90.8%, 86.0%). Differences of accuracy were found for the four size levels (*p* < .05 with one-way ANOVA on factor size level), so were the differences of RTs. For pairwise t-tests between groups, differences of accuracy were found between *small* and two larger size levels (both *p* < .001), as well as between *smallest* and the other three larger size levels (all *p* < .001). The same discriminative patterns were observed for RTs.

**Fig 5:**
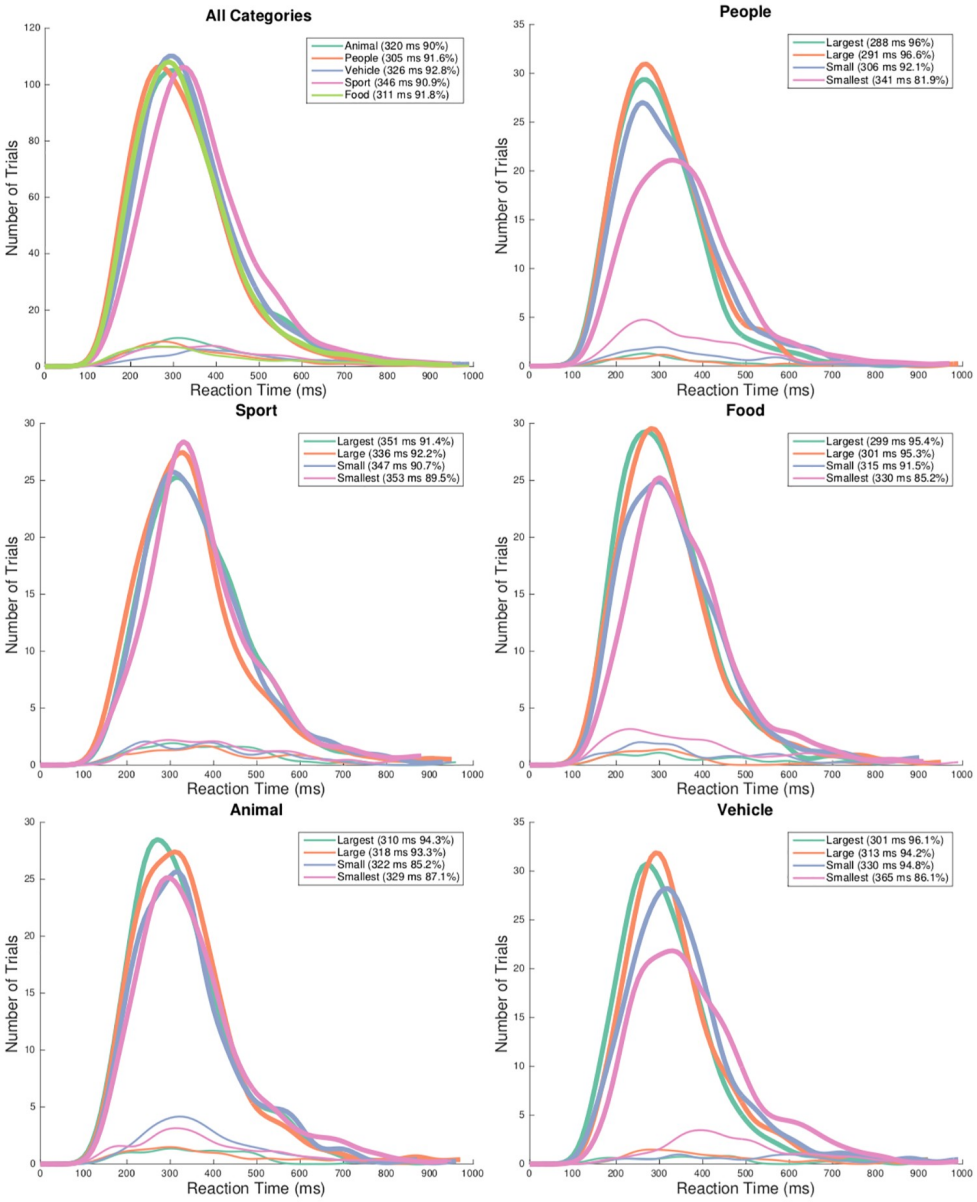
RT distributions for target objects at different levels of scales. [The top-left sub-figure] RT distributions for correct (thick line) and incorrect (thin line) responses for each target category with the percentage of responses pooled across all subjects and time characterized with 10 ms time bins (regardless of scales). Access to processing the *people* category was faster than the others (s.). [The other sub-figures] RT distributions with five categories as targets respectively. The time-course and accuracy of processing *sport* category were both similar across scales (t-test both n.s.), while the performance was variant with the change in scales with other four categories as targets (t-test both s. for other four categories). Note that (n.) s. refers to (no) significance in the t-tests.

With four levels of target object display size, target categories at each individual scale level were further analyzed (Fig 5(B)–5(F)). No differences in terms of accuracy and speed were found for *sport* towards factor size level (*p* < .01 with one-way ANOVA on factor size level). Each of the other categories showed different performance in terms of accuracy and speed towards the change in display size level (for each category, *p* < .01 with one-way ANOVA on factor size level).

**Fig 6:**
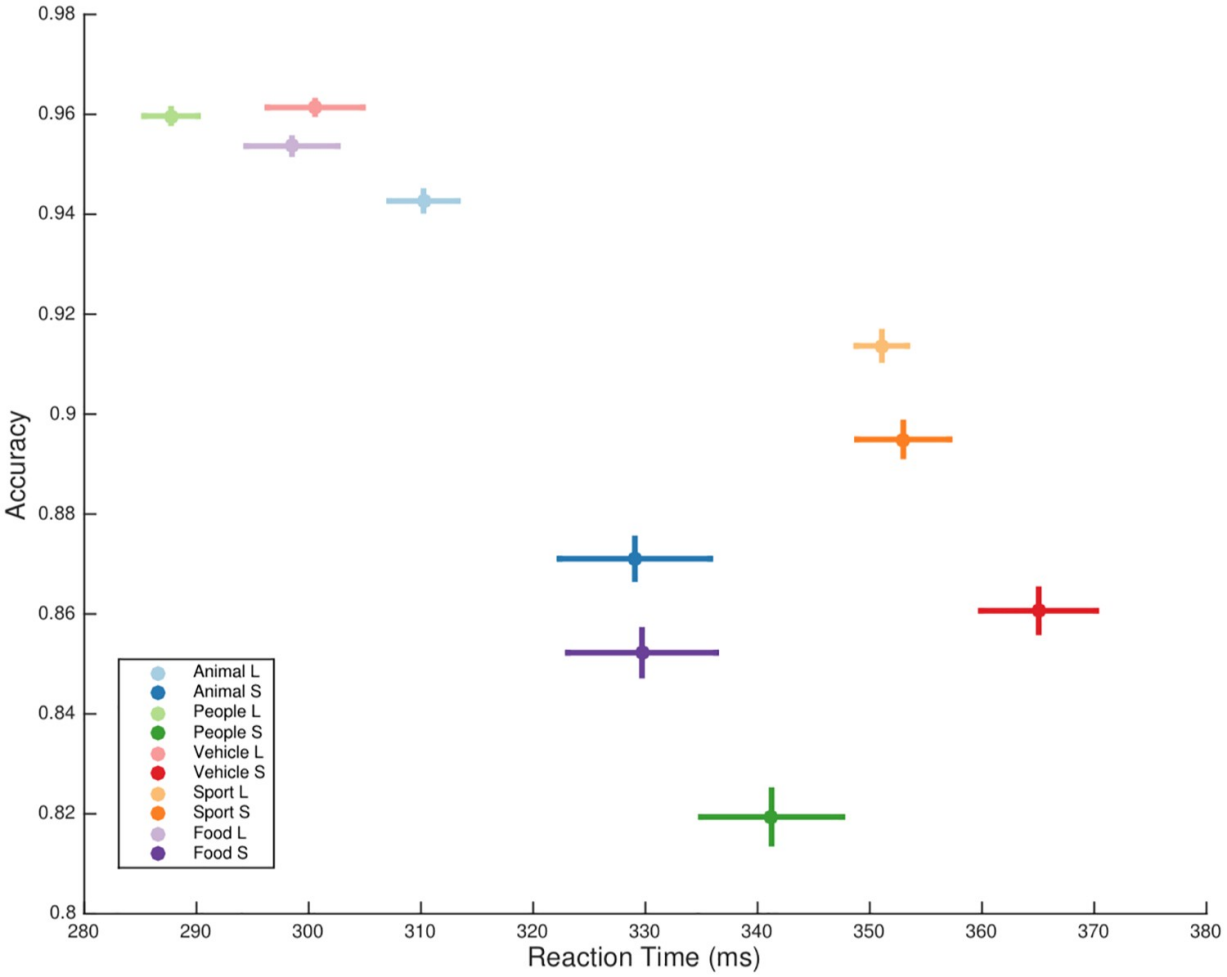
Accuracy vs. median RT for target objects at two levels of scales. To facilitate the comparison among the performance of different category targets, two extreme scales were used in this analysis: “L”—the largest level, “S”—the smallest level. Horizontal and vertical bars indicated standard error for median RTs and accuracy respectively. The values were computed using a bootstrap method that divided the samples into 10 parts.

For clarity reasons, Fig 6 plotted accuracy vs. RT for the five categories at the *largest* and the *smallest* display size levels. By inspecting two groups with two size levels, the decreased accuracy and the increased RTs towards decreased target size were consistently observed in each of the four categories excluding *sport*.

### Comparisons between human and model performance

In order to see if the patterns of results could be accounted by standard computational models, we assessed the performance of two models: SALIENCY model [56] and GIST model [51], both with SVM classifiers. They both showed success in explaining certain characteristics of the fast feedforward visual processing [7, 11], and were complementary in that SALIENCY focused on low-level and local information while GIST on global statistics. They were tested on the identical image sets used in the human experiments (see Materials and methods for details). In contrast to the previous two models that were to explain the human performance, we also compared recognition accuracy from human observers and the state-of-the-art object detection model that mimicked the human prior experience via a large amount of training data.

**Fig 7:**
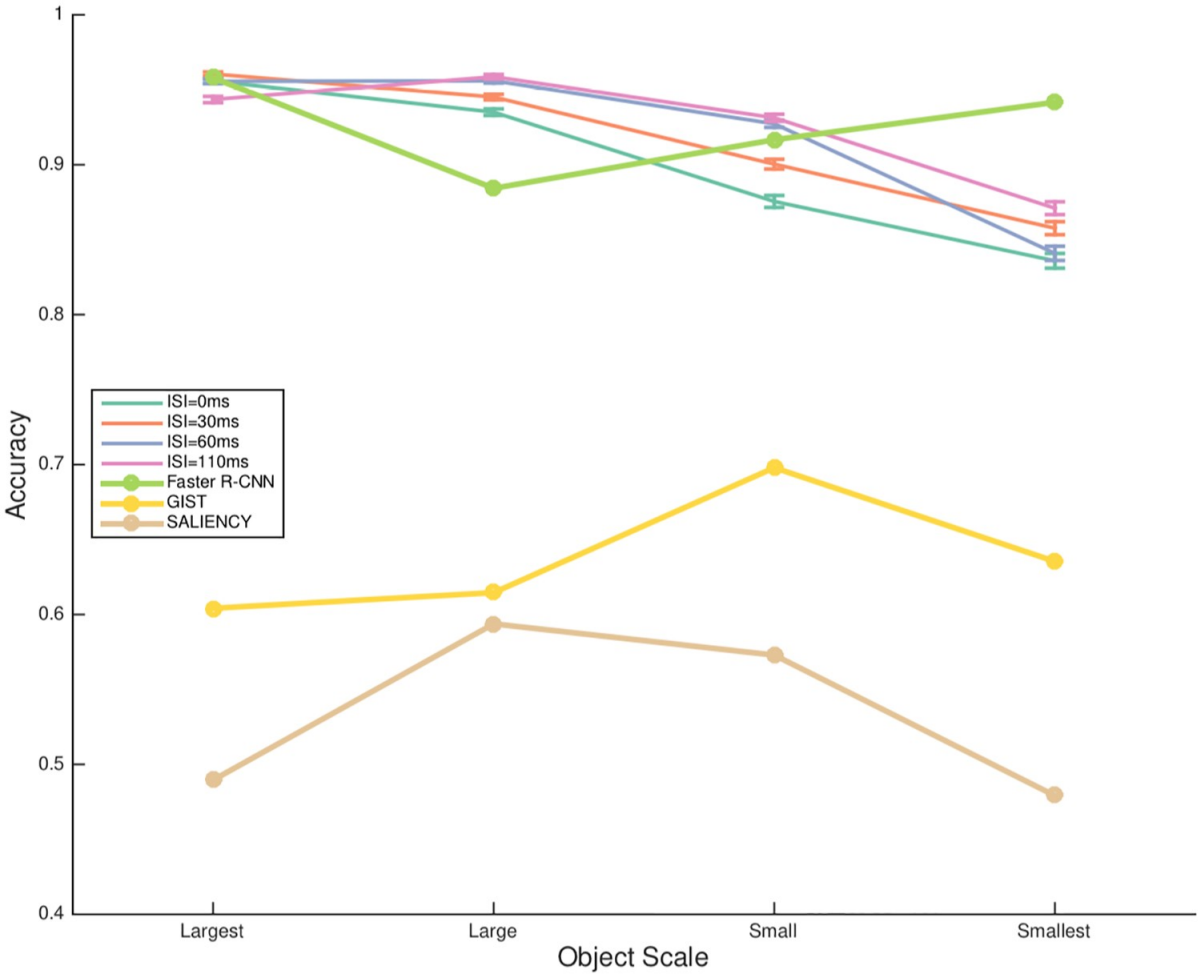
Comparisons in performance between standard computational models and human observers with regard to scales and ISIs. These results were averaged from four target categories except the *sport* category due to the special categorization of scale levels for this category. Two standard computational models SALIENCY and GIST that are believed to account for information conveyed during the first feedforward sweep could not reach human performance. Faster R-CNN built on backbone with FPN and ResNet-50, showed competitive recognition accuracy to human performance.

As shown in Fig 7, the accuracy of human performance decreased as the scale of target objects decreased from the *largest* to the *smallest* (*p* < .05 with factor size level), at each of four levels of the inter-stimulus interval (ISI) values. Recognition accuracy by human observers decreased when ISI got shorter significantly (*p* < .05) with the *small* scale of target object, while showing no significant trend with each of the other three levels of target object (at each size level, *p* > .05 with factor ISI). While the GIST model performed better, the SALIENCY model only performed nearly at a chance level which showed that the low-level saliency information did not account for the behavioral results, and thus validated the stimuli [7]. As for the state-of-the-art object detection model i.e. Faster Region-CNN (R-CNN) built on backbone with ResNet-50 and Feature Pyramid Network (FPN) [45, 53], it is shown to perform similarly to human behavior in terms of recognition accuracy rate except that it largely outperformed human’s performance on the *smallest* targets. This is because humans have to trade-off between the accuracy and speed in the rapid recognition task, leading to the varying performance across the scales, in line with previous finding for human rapid recognition performance [37]. In contrast, the FPN module makes the object detection model robust to recognition at all scale levels of target objects.

Note that Fig 7 was from blocks targeting four of the five categories except for *sport*, since the definition of scales of the *sport* category was based on the size of the interacting *people* rather than the equipment, different from the definition of scales of other four categories (see Methods and materials for details). For the *sport* category, ANOVA test revealed that human performance showed no difference with regard to scale at each of the four levels of ISI (n.s.), and the modulation of ISI did not affect accuracy over all scale levels (n.s.), meaning more robust performance towards the changes.

**Fig 8:**
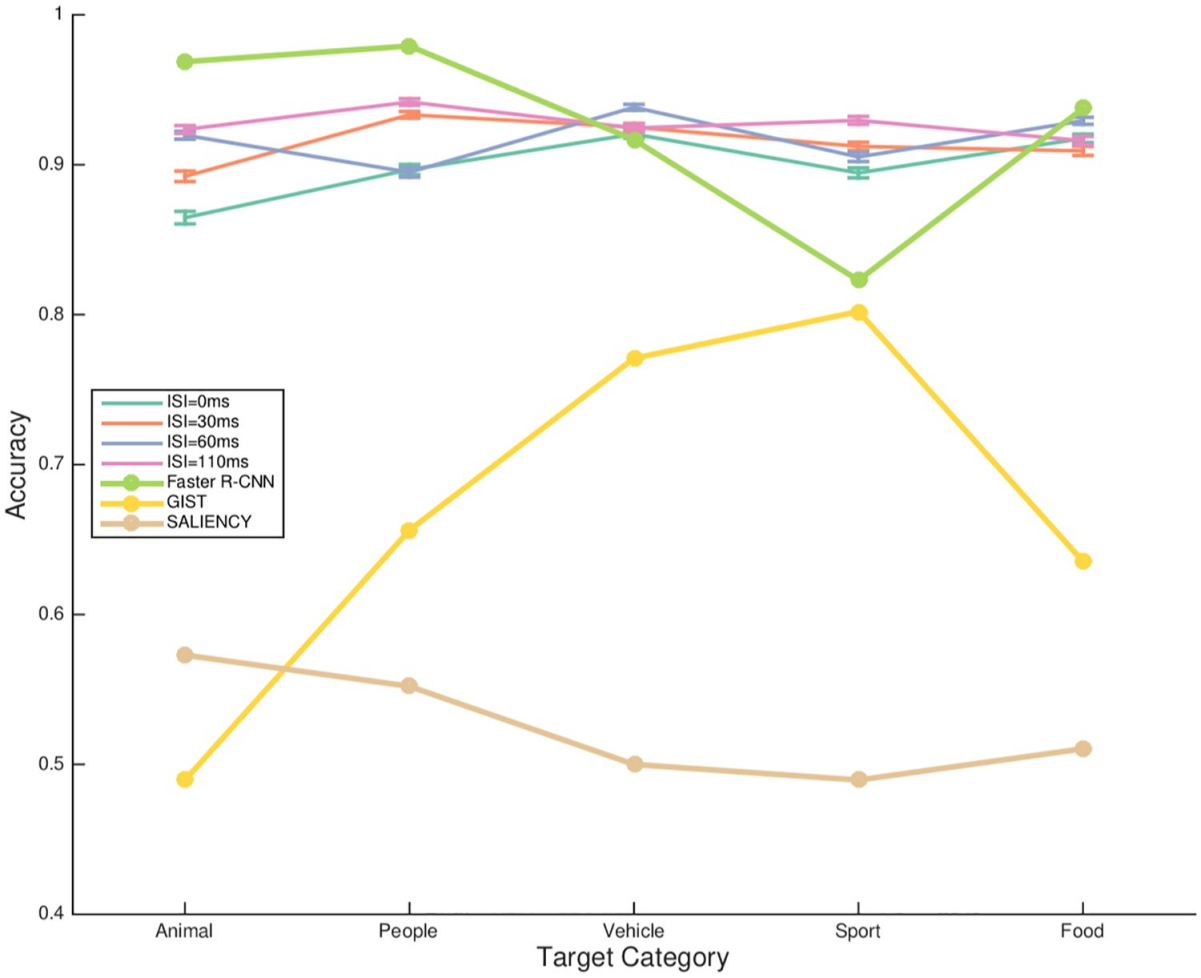
Comparisons in performance between standard computational models and human observers with regard to target categories and ISIs. Results at four levels of ISI values were showed for human observers. Three standard computational models (i.e. SALIENCY, GIST, Faster R-CNN built on FPN and ResNet-50 backbones) were also employed for auxiliary investigation.

Fig 8 compared rapid recognition accuracy rates from human observers and the computational models across categories. In general, humans performed consistently well on the five categories. Faster R-CNN performed better than or equivalently to humans on recognizing *animal*, *people*, *vehicle* and *food*, as they learn the discriminative features from abundant training of recognizing common categories. Yet, it performed worse on recognizing *sport* than humans. Interestingly, while GIST and SALIENCY were not as competitive in classification accuracy rate, GIST performed almost close to Faster R-CNN on recognizing *sport*.

**Fig 9:**
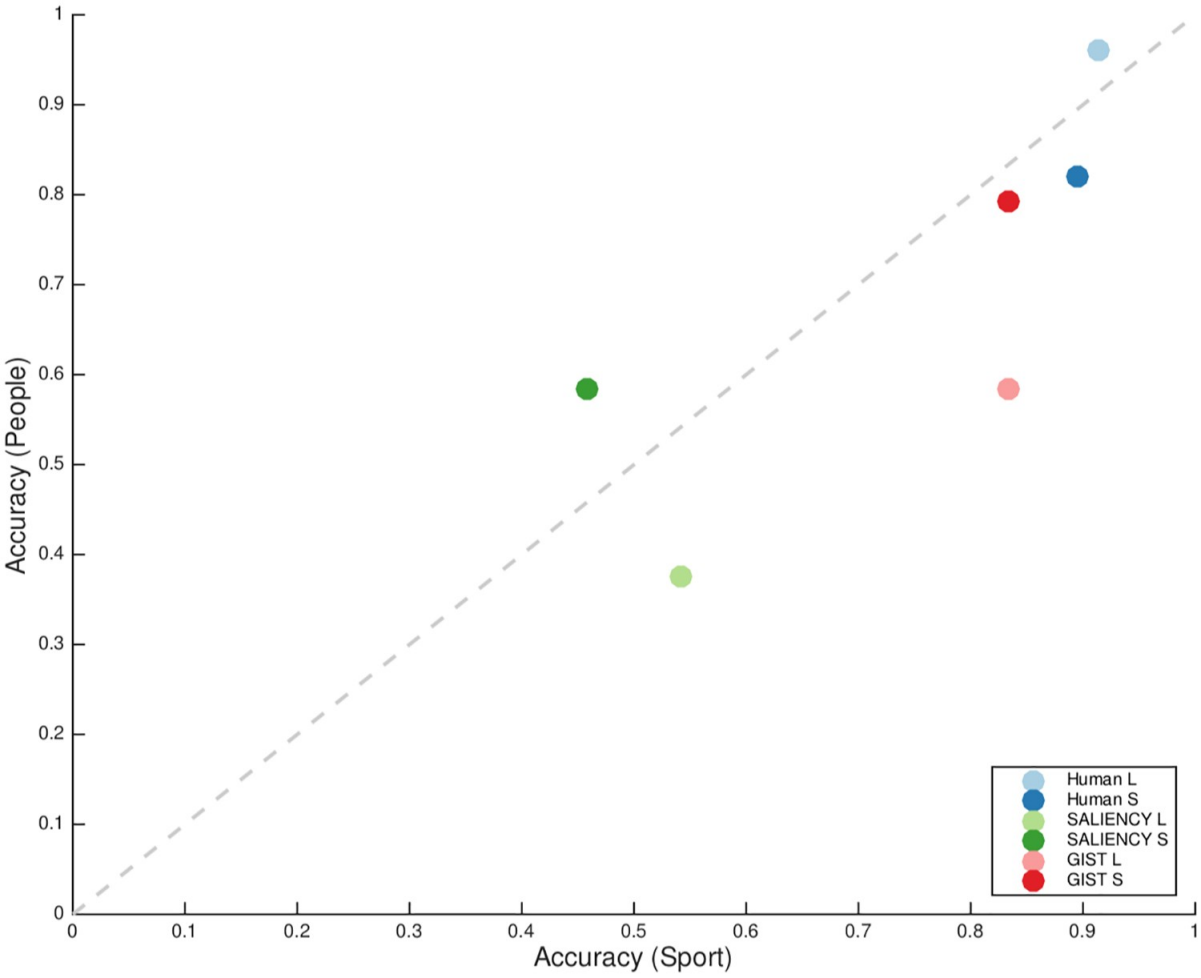
Comparison in performance between standard computational models and human observers towards the recognition pattern of people and sport categories. Again, two scales are analyzed here: “L”- the largest level, “S”—the smallest level. SALIENCY model performed at a chance level that validated that low-level cues were almost not informative in the dataset. GIST model that represented global layout statistics was close to explaining human data when the target object (the human-object pair for *sport* category) is relatively small.

To understand the performance towards *sport*, we further conducted a comparison between the trials targeting *sport* and *people* from both humans and the two standard computational models (Fig 9). The SALIENCY model performed at a chance level in either the largest or the smallest scale level of target objects, which again showed that low-level clues are not informative in the image sets. The performance of the GIST model was close to human performance when the target was of small scale but still had a gap when the targets were relatively large. Another interesting result was that, in contrast to the performance of human subjects, the GIST model scored higher correct rates when targeting *sport* than targeting *people* in the images at both object scale levels.

**Fig 10:**
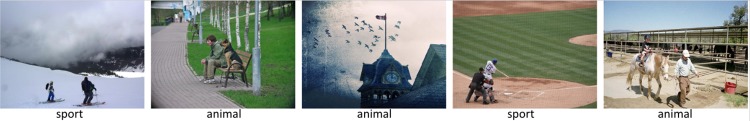
Qualitative evaluation between humans and computational models. Five images with the least recognition rate by human observers are displayed, respectively. The target categories are mainly *sport* and *animal*, with the smallest display size. In contrast to human performance, computational models are able to correctly recognize the respective categories in most of these images.

To further understand the human vs model comparison, we qualitatively showed and analyzed the error patterns in example images shown in Fig 10. Five images with the least recognition rate by human observers were displayed, respectively. These images revealed common recognition error patterns by humans. We observed that the potential causes for poor recognition rates could be due to the confusion between target and the contextual objects, the ambiguous actions to recognize *sport*, and the small scales for capturing object attributes. In contrast to humans, Faster R-CNN recognized all five images correctly that seemed hard for humans. While correctly recognizing images in the first three columns in Fig 10, GIST failed on the rest two images and SALIENCY failed on the fourth image where the target object of the respective category was of ambiguous color and shape to the contextual objects.

## Discussion

The experiment results aimed to reveal the combination of top-down influences associated with task demands by varying animacy, level of abstraction, display size in the ultra-rapid categorization task. We found that all of these manipulations influenced ultra-rapid categorization processes. This has an impact on the interpretation of the extent of the top-down effects involved in the ultra-rapid processing.

### Level of abstraction between “people” and “people playing sport”

This study extended previous studies from recognition of human faces and body to *people* [31, 57]. Importantly, the comparison in Fig 3 between *people* and *sport* supported the superordinate advantage in ultra-rapid categorization [15] with specific examination of people, where *people* is at the superordinate level of abstraction.

#### Temporal advantage

One possible explanation for the quick access of the *people* category includes two aspects. First, when the target scale is large thus the human faces are clear, recognition advantage of human faces dominates. That is, the temporal advantage towards *people* could be due to the human face processing advantage, involving a special processing module possibly tuned by a great deal of expertise in interaction with people from an early age [58] or by evolutionary priorities [7]. Second, when the target scale is relatively small, motion plays a central role in recognizing *people* [57, 59, 60]. The primacy and prominence of body motion cues to identity at a distance have been validated [61, 62], with the face becoming less resolved. It has been validated that human can quickly recognize body and body parts with the extrastriate body area (EBA), a sub-part of the extrastriate visual cortex [34].

Another interpretation may come from the dual process approaches in visual recognition [63]. One approach is fast based on the holistic features, while another one is slower using the structural description representation. Since the stimuli included a wide range of different exemplars, sizes, and eccentricities, it is difficult to quantitatively examine the two ways. We speculate that visual processing of *people* involves fast recognizable holistic features.

However, in the complex naturalistic scenes, the factors such as position, viewpoint, ambiguity of surrounding context of the target objects in the images were highly variable, mimicking real-world viewing conditions, thus they might possibly lead to different recognition patterns from recognizing isolated targets with homogeneous background. This could possibly account for the inconsistent speed advantage in recognizing “people” at different levels of scales.

#### Contextual effect of “people”

It remains elusive to what extent *people* interacting with equipment modulates recognition of *sport* activities. We speculated that scale of *people* may influence the contextual effect. We performed computational modeling of scene representations. In contrast to the human performance, the GIST model was slightly more accurate at discriminating *sport* as target than *people* as target regardless of the scale levels. Also, the GIST model explained the performance with *sport* and *people* categories well when they were relatively small in a scene, but not with large targets (Fig 9).

On the one hand, for the largest scale of targets (here refers to the *people* in both tasks), rapid processing of either *people* or *sport* could not only be based on early access to global scene statistics. Rather, the results indicated that in such case, rapid recognition of *people* and *sport* might be based on global scene statistics, as well as an extraction of specific semantic category features of human, such as human face, pose and body motion [31, 34, 57]. On the other hand, for the smallest scale of targets, spatial layout properties managed to account for the processing of both *people* and *sport* activities. This is easily expected that the small scale of *people* categories dissipated the influence from the categorical features. Instead, the global layout features dominated the recognition of the targets.

In summary, *people* interacting with objects might impose contextual influences on the processing of the objects if the *people* was of enough large size in the scene. Otherwise, global spatial representatives of the images dominated the processing.

### Real-world size

In contrast to the drastic decrease in accuracy from recognition of the largest to the smallest *people* category, human subjects scored consistently high correct rate with *sport* as targets at all levels of display sizes (Fig 6). The same pattern of robustness was also observed in accuracy towards ISI change between targeting *people* and *sport* (Fig 8). These evidence might imply the involvement of top-down processing demanded for association of object real-world size and its display size. Note that the sizes of the *people* interacting with the *sport* category were matched to the ones in images with *people* as target.

As we observe, a more specific set of visual features are required for recognizing *sport* than *people*. For example, more visual detail is needed to detect whether there is “a person playing sport” rather than just “a person”. It might indicate that increasing accumulated visual information could ensure the invariance of performance. This view is validated by Bacon *et al*. [37] that enough accumulated visual information ensured the invariance towards ISI change by manipulating enough long stimulus duration in the backward masking paradigm. These results may also imply that a longer RT to target *sport* allowed it to get higher levels in the processing pathway, thus better supporting invariance in performance towards scale [64] and ISI change. This view is supported by the finding that from human V1 to IT area in visual processing pathway, there is an increase in invariance to position and scale [38]. As one important aspect of top-down processing is the association of object real-world size and display size [15], the robust performance towards change of size in *sport* provides cues that top-down/feedback processing is likely to be involved in such rapid processing.

### Effect of animacy from “animal”, “vehicle” and “food”

The comparisons between *animal* and *vehicle* in Fig 3) replicated the results in previous literature [55] that RTs were not significantly different between rapid recognition of *animal* and *vehicle* that represented animate and inanimate categories, respectively. We extended to another socially important category *food*. We found that *food* as an inanimate category, was categorized faster than *animal*, while showing comparable accuracy of performance. We also noticed that *food* showed robust accuracy towards the change in ISI, which controlled the accumulated amount of information.

This interesting finding possibly indicates that an easily accessed and reliable visual information template resides in categorizing *food*. This implication is based on the evidence that it is categorized fast and accurately even when given a very brief duration for processing information accumulation. Indeed, *food* has been found to associate closely with human life, thus inducing recognition advantage [27]. It indicates an open future direction about the perceptual feature template for ultra-rapid categorization of *food*.

### Human vs. computational model object categorization

Based on the comparisons in rapid recognition performance between humans and the three computational models (see from Figs 7 to 10), we can learn from several perspectives. First, Faster R-CNN generally outperformed humans in the rapid object recognition task, indicating that discriminative information for the model was in place but humans had to trade-off decision making speed and the extracted information [41, 65] (see Fig 7). The scaling invariance analysis showed that in contrast to humans’ varying recognition accuracy with respect to target scale in the demanding ultra-rapid scenario, Faster R-CNN performed robustly. This again validated the trade-off between speed and accuracy by humans in the rapid recognition task, and the task became more demanding when the target got smaller. Yet, Faster R-CNN extracted the discriminative features regardless of time. While Faster R-CNN performed well in general across various target object scales, and various target categories, there was an exception that it recognized less accurately than humans on the *sport* category (see Fig 8). This notable observation was possibly because the training samples of *sport* for the Faster R-CNN only annotated the equipment for object recognition, which was often small in naturalistic scene images. However, humans could better infer *sport* based on other information in the scene such as human actions and human clothing, enhancing the recognition accuracy. Explicit addition of these representations, that humans often use, may increase recognition accuracy of models.

Second, while the performance of GIST, SALIENCY models was far from human performance over rapid object recognition on naturalistic scenes, GIST scored high on recognizing the *sport* category (see Fig 8). Consistent with the literature [38], feedforward models such as GIST and SALIENCY with the low-level image features were insufficient in representing objects in complex real-world scenes. GIST, a model representing global scene statistics performed close to Faster R-CNN on targeting the *sport*. This observation indicates the importance of global scene layout in recognizing complex category such as *sport* which complements the local object features for recognition.

Third, from Fig 10, we observed that the potential causes for low recognition rates by humans could be the confusion between target and the contextual objects, the ambiguous poses to recognize *sport*, and the small scales for capturing object attributes. In contrast, the computational models could correctly recognize most of these images that were difficult for humans. It suggested that the generic mechanisms of the examined machines and humans to extract discriminative features for object recognition were different, so were the error patterns. Faster R-CNN, trained on copious number of naturalistic images with objects annotated could overcome the error from visual ambiguity. For example, it could correctly recognize the *dog* when both people and dog sitting on the bench. GIST and SALIENCY, representing the global and local low-level features respectively were susceptible to this error pattern as humans were. It indicated that for ambiguous scenes, the human visual process was not more efficient than using the low-level visual cues.

Finally, human studies have been commonly designed for specific purposes and with specific hypotheses. It is therefore non-trivial to adapt them into suitable models for comparisons. Our work that enabled such a comparison makes a concrete step toward this goal and may shed light for possible collaboration or integration of the two parts that is of interest to both communities.
